# Chronic tobacco smoking and neurocognitive impairments in adolescents and young adults: a systematic review and meta-analysis

**DOI:** 10.3389/fpsyt.2024.1384408

**Published:** 2024-04-23

**Authors:** Ahmed Elatfy, Sebastian Vrahimis, Aldo Conti, Alexander Baldacchino

**Affiliations:** ^1^ School of Biotechnology, Nile University, Sheikh Zayed City, Giza, Egypt; ^2^ School of Medicine, Ninewells Hospital, University of Dundee, Dundee, United Kingdom; ^3^ School of Medicine, Division of Population and Behavioural Science, University of St Andrews, St Andrews, United Kingdom; ^4^ Department of Child & Adolescent Psychiatry, Institute of Psychiatry, Psychology and Neuroscience, King’s College London, London, United Kingdom

**Keywords:** nicotine, chronic smoking, tobacco, neuropsychology, neurocognitive impairment, adolescents, young adults, systematic review

## Abstract

There is a lack of robust research investigating the association between neurocognitive impairments and chronic tobacco smoking in adolescents/young adults. Therefore, a systematic review and meta-analysis were conducted to examine this association by pooling cross-sectional studies published from 1980 to 2023. The systematic review assessed the neurocognitive performances between chronic tobacco smokers and non-smokers in each study. The meta-analysis included six studies that compared chronic tobacco smokers against non-smokers using neuropsychological tests covering three neurocognitive domains. The results showed a cross-sectional association between impairpments in *motor impulsivity* across two aspects: *reaction delay* and *incongruent errors*, with the effect size being (SDM = 0.615, p = 0.000) and (SDM = 0.593, p = 0.000) respectively. However, no significant associations were found for *intelligence* (SDM = 0.221, p = 0.425) or *working memory* (SDM = 0.150, p = 0.581). This study highlights the need for further research to explore a greater number of neurocognitive domains in the context of chronic smoking in adolescents/young adults, particularly motor impulsivity, intelligence and working memory, as well as the socioeconomic factors involved. There is also a need to further study the effects of emerging alternative nicotine administration methods in this age group.

## 1 Introduction

Chronic tobacco smoking, defined as daily cigarette smoking (>10 cigarettes per day) for 2 or more years, is considered by the World Health Organization (WHO) as significantly hampering effective global public health interventions (1). It is estimated that in 2019 there were 155 million individuals across the world aged 15-25 years who were smoking tobacco (2). Across all populations, smoking contributes to over 8 million deaths around the world each year, either directly or indirectly, and global tobacco consumption is a contributing factor to 7 million deaths per year, with around 1.2 million non-smokers dying from second-hand smoking each year (1).

In 2008, nicotine was identified as the most addictive substance across the world, with smoking tobacco a major cause of cardiac and respiratory disease (3–5). Data from the Health Survey for England show that in 2021, 13% of young people (16-24 years old) were current tobacco smokers, while the highest prevalence of smoking is between the ages of 25-34 years, at 18% (6). These rates can be compared to data from the Office for National Statistics (ONS), the UK’s national statistical institute, which show that in 2021, the overall rate of smoking is 13.3% in people over 18 years of age (this figure reaches 21.1% in Scotland) (7, 8). However, the prevalence of chronic tobacco smokers in the UK has been decreasing since 1974, though over recent years this may be partially linked to the increasing popularity of electronic nicotine delivery systems (ENDS, also known as vapes or e-cigarettes) as an alternative source of nicotine, as well as variations in behaviour during the COVID-19 pandemic (8). Notably, the use of ENDS is most popular in the 16–24-year age group (8). Importantly, the number of deaths related to smoking remains high, with 74,600 recorded in England over 2019 – the most recent years for which data are currently published by National Health Service (NHS) Digital (9). Chronic tobacco smoking is still a significant behaviour among adolescents and young adults, demonstrating the importance of reducing the number of young people who smoke (10).

Chronic tobacco use often begins during the adolescent phase of life, with 90% of smokers beginning before the age of 18 years (11, 12). Additionally, the younger an individual begins smoking the harder it is to quit (13). Numerous studies indicate that individuals who begin smoking tobacco in their early life (<16 years old) have a higher probability of becoming chronic tobacco smokers, and developing an addiction to nicotine, in comparison to individuals who have a later onset of smoking (>16 years old), again contributing to the difficulty in quitting smoking once in adulthood (14–18). As a result, reducing the number of adolescents and young adults who start smoking would likely impact the total number of chronic smokers over time.

The transitioning phase between childhood and adulthood, known as adolescence, is characterized as a learning phase that includes behavioural changes, such as elevated levels of risk-taking behaviour, seeking novel experiences, and independence (19–21). According to WHO, the period of adolescence ranges from 10 to 19 years old. However, other studies have proposed that adolescence lasts until 25 years of age which can also be called the young adulthood phase, based on the brain’s ongoing maturational processes (22). This ongoing maturation or “rewiring” of the brain is known to be governed by numerous specific stages of physical, emotional and cognitive maturation, and, as reported by Gavin et al. (23)Arain et al. (, 24), and Sylwester (25), it is known to start from around puberty, at the age of 10 years, until the brain reaches the stage where it is most mature at the age of 24 years.

A key consideration in the context of tobacco smoking during adolescence is the association this may have on an individual’s neural development. Central nervous system (CNS) development begins in the third week of gestation and through to late adolescence, regulated and coordinated through complex cellular, genetic, and environmental factors (26). During the adolescent phase of life, the human brain is undergoing numerous neurodevelopmental transition and maturation processes (27). It is the phylogenetically more recent cortical regions of the CNS that demonstrate the ongoing and prolonged development through childhood and into adolescence (28). This is important to consider in the context of chronic tobacco smoking in this age range, as the development of these central neural regions underpins emotional, cognitive, and behavioural changes seen in adolescence (28).

Chronic exposure to nicotine during adolescence has also been shown to be associated with an increase in the probability of an individual developing major psychiatric disorders and neurocognitive impairments in later life. Most commonly, adolescent, and young adult chronic tobacco smokers experience a level of progressive attentional deficit (29). Specific neurocognitive disturbances seen in studies include changes to working memory and attention, with a notable reduction in the activation of the prefrontal cortex (PFC) (30, 31). There are also specific psychiatric conditions that are associated with chronic nicotine exposure in adolescence, including major depressive disorder, schizophrenia, and addiction to other substances (32–38).

In summary, there is a need for clinical research to improve the understanding of the complex relationship between chronic tobacco smoking and neurocognitive impairments in individuals from younger age groups, as suggested in a previous systematic review and meta-analysis in adult populations (39). Using restricted inclusion criteria for the age groups (10-24 years old) of participants assessed (23–25), the following is a systematic review and meta-analysis of the existing studies on chronic tobacco smoking and neurocognitive impairments in adolescents and young adults.

## 2 Methods

This review was done in compliance with the Meta-analysis of Observational Studies in Epidemiology (MOOSE) guidelines (40) and the Preferred Reporting Items for Systematic review and Meta-Analysis (PRISMA) guidelines (41) (**Supplementary Table 5**). The study protocol registration was made on the PROSPERO database (CRD42023428359).

### 2.1 Literature search

#### 2.1.1 Inclusion and exclusion criteria

The PICO criteria utilised in this review were (1) studies including human participants (2) with ages ranging from 10 years to 24 years (23–25), (3) experiencing chronic tobacco use as defined by the WHO (1) and (4) including all types of studies. As for the comparison group, they were defined as healthy participants who do not smoke (nicotine naïve), of the same age group (10-24 years of age). Furthermore, these papers had to supply the name of the neurocognitive tests used and which neurocognitive domains (e.g., Impulsivity, Attention, Memory, etc.) were being assessed during each test (42). Chronic tobacco smoking was defined as daily cigarette smoking (>10 cigarettes per day) for 2 or more years.

The exclusion criteria used were as follows:

(A) Cohorts employing participants with illicit poly-drug use and/or dependence.
(B) Cohorts employing individuals with more than 14 units of alcohol per week as the alcohol cut-off.
(C) Cohorts employing individuals diagnosed with neurological illness and/or any Axis 1 Psychiatric Illness (DSM IV/V).
(D) Studies that had no healthy comparator group (non-smoker controls).
(E) Studies not utilising neurocognitive tests.

#### 2.1.2 Search terms

The search terms utilised were: (Nicotine OR Cigarettes OR Tobacco OR ‘Chronic Smoking’) AND (‘Neuropsychological impairments’ OR ‘Cognitive impairments’ OR Neurocognition) AND (Adolescents OR Teens OR ‘Young Adults’).

Next, the search terms ‘neuropsychological impairments’, ‘cognitive impairments’, and ‘neurocognition’ were replaced by the names of the specific neurocognitive tests. These were: ‘Rapid Visual Information Processing’, ‘Wechsler Adult Intelligence Scale’, ‘Spatial Working Memory’, ‘Ray Auditory Verbal Learning Test’, ‘Two Back Test’, ‘Trail Making Test’, ‘Stroop Test’, ‘Wisconsin Card Sorting Test’, ‘Stroop Colour Word Task’, ‘Reaction Time’, ‘California Verbal Learning Test’, ‘Verbal Fluency’, and ‘Gambling Test’ (42).

#### 2.1.3 Search engine

The literature search was conducted in May 2023 using the following databases: PubMed (1980-2023), APA PsycINFO (1980-2023), Cochrane Central (1980-2023), SciELO (1980-2023), and Scopus (1980-2023). Two further studies were located using Google Scholar. All the identified studies from the database search were reviewed and moderated by the authors for the selection of eligible and suitable papers to be used for this systematic review and the meta-analysis. Finally, to improve the comprehensiveness of the identified studies, the references of the accepted studies were reviewed, and a “snowballing” technique was employed.

Three authors (AE, SV, and AAC) screened the studies independently using the inclusion and exclusion criteria listed above. First, the title/abstract of the studies was screened. This utilised EndNote 20, from which search libraries were uploaded to Rayyan. Rayyan software was then used during the screening process. Subsequently, the full text of the articles that passed the title/abstract screening was inspected. Disagreements were resolved consensually.

### 2.2 Analysis

#### 2.2.1 Qualitative analysis

Several papers were reviewed to further investigate the effect of chronic tobacco smoking and neurocognitive impairments in adolescents and young adults. Neurocognitive impairments were pooled from each paper. Then, these findings were compiled in a descriptive summary to be further investigated and used in a preliminary conclusion for the neurocognitive impairments that can be associated with chronic tobacco smoking in adolescence or young adulthood.

#### 2.2.2 Quantitative analysis

##### 2.2.2.1 Data extraction

This was followed by meta-analytic calculations to reach a quantitative estimate of the impact of chronic tobacco smoking on the neurocognitive functions of the identified cohort. Means (M) and Standard Deviations (SDs) of scores on neurocognitive tests/measures were extracted from six studies (5, 18, 21, 30, 43, 44) and inserted into the Comprehensive Meta-Analysis (CMA) version III software package for analysis (45). It was only possible to extract data from six studies as the other research papers pooled for the systematic review did not provide relevant statistical data. Data were limited to three neurocognitive domains: Motor Impulsivity, Intelligence, and Working Memory. These domains were identified from the neurocognitive tests utilised by the studies included in the review following a previous meta-analysis by Conti et al. (39) and Figueiredo et al. (46), and the guidelines of Baldacchino et al. (42) (**Supplementary Tables 1**–**3**). Regarding Motor Impulsivity, the data extracted included those pertaining to the Stroop Task ‘response delay’ outcome measure (measured by reaction time during the incongruent condition minus reaction time during the congruent condition) and Stroop Task incongruent errors.

##### 2.2.2.2 Meta-analysis

A random effect model was selected to conduct meta-analytic calculations instead of a fixed effect model as it was assumed that the pooled studies were not ‘identical’ (i.e., not displaying the same true effect size) (47, 48). The ‘Standard Mean Difference’ (SMD) was selected as a statistical summary measure. Effect sizes were computed utilising Cohen’s benchmark criteria; an effect size of 0.8 would have implied a ‘large’ effect size, an effect size of 0.5 would have implied a ‘medium’ effect size, and an effect size of 0.2 would have implied a ‘small effect size’ (49). Heterogeneity was assessed by using both Cochran’s Q and I2 tests (47). It was not possible to run a meta-regression by utilising relevant smoking characteristics of participants as moderators (e.g., number of cigarettes smoked x day, pack-years) due to the low number of studies (<10) pooled for each neurocognitive domain (47).

#### 2.2.3 Publication bias

Publication bias refers to the tendency to publish studies reporting statistically significant results than studies reporting results that are not statistically significant (50, 51). Therefore, there is a possibility that studies included in a meta-analysis would be biased and consequently reflected in the results of the quantitative synthesis (39). Publication bias for the studies included in the meta-analysis was assessed through the visual inspection of Funnel’s Plots (47).

#### 2.2.4 Assessment of study quality

To evaluate the quality of papers that were included in the review, the National Institutes of Health (NIH) case-control quality assessment tool was utilised (52). Using the Study Quality Assessment Tools, the studies were either classified as ‘poor’ indicating that the study in question presents a high risk of bias, ‘fair’ indicating that the study in question presents a moderate but not to the extent to invalidate the results, or ‘good’ indicating that the study in question presents a low risk of bias (52).

## 3 Results

### 3.1 Search results

**Figure 1:**
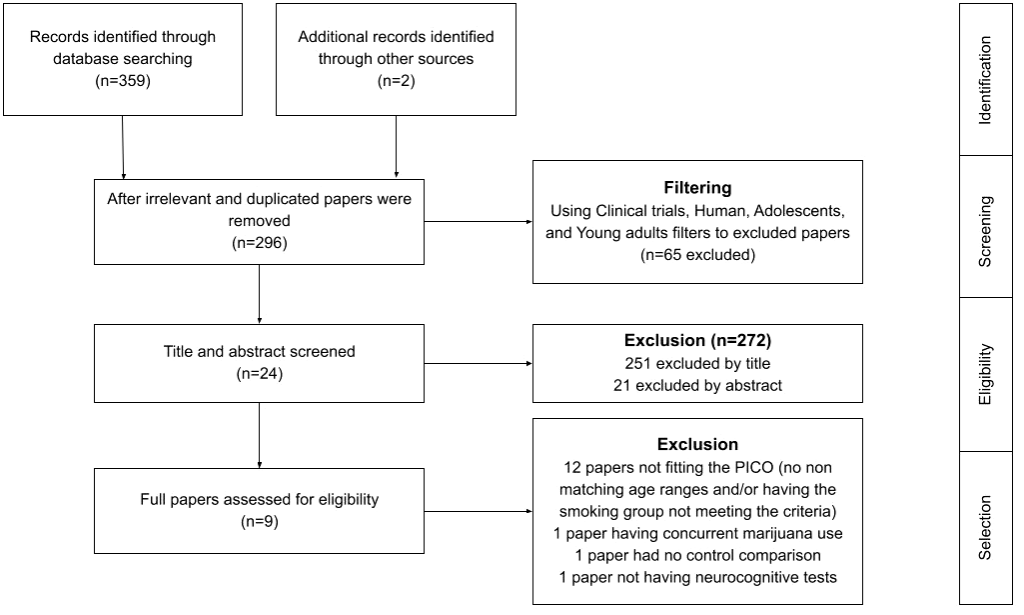
Neurocognitive associations of chronic smoking on adolescents/young adults.

Initially, a total of 359 papers were identified. Then filtering tools on these databases were utilised to filter for the following: ‘Clinical Trials’, ‘Human Trials’, ‘Adolescents’, and ‘Young Adults’. The citations were downloaded to EndNote20 and then uploaded together to Rayyan. Rayyan is an online software program designed specifically for researchers working on systematic literature reviews, which has tools that improve the organisation and efficiency of the screening and selection process of studies. Duplicate papers were removed manually by AE and SV, using Rayyan software to assist the process, excluding 63 duplicates and leaving 296 remaining unique studies. Titles and abstracts were then inspected to assess the studies for eligibility by AE, SV and AAC. This inspection process resulted in the exclusion of 272 papers; 251 papers were excluded by the title, and 21 were excluded after reading their abstracts. Next, using the inclusion and exclusion criteria, the remaining 24 papers were reviewed comprehensively for eligibility by AE, SV and AAC, which yielded the elimination of 12 more papers due to having non-matching age ranges and/or having the smoking group not meeting the criteria of inclusion. One paper was excluded due to concurrent marijuana use, one paper was excluded that had no control (non-smoker) comparison, and one paper was excluded that did not include neurocognitive tests. Eventually, this yielded 9 case-control studies that were selected to be included in the quantitative synthesis (**Figure 1**).

One study included in the quantitative synthesis reported data from an additional comparator group (‘Light Smokers’) (53). Therefore, to comply with the exclusion and inclusion criteria and the aim of this study, only the appropriate comparator groups were included in this meta-analysis.

The studies included in the analysis originated from four countries, including the one from United States of America (30), five from China (5, 18, 21, 43, 54), two from Saudi Arabia (44, 55), and one from Belgium (53).

The quality of the studies was assessed consensually by AE and SV (**Supplementary Table 4**). Out of the nine studies that were accepted for inclusion in this current meta-analysis, three were classified as ‘good’ and six were classified as ‘fair’ (**Table 1**; **Supplementary Table 4**).

### 3.2 Sociodemographic

Demographic data were utilised from a total of (307) chronic tobacco smokers and (315) non-smoking controls, all free of any neuropsychiatric disorders. Since adolescents and young adults were the targets for this study, the mean age range of the adolescent and young adult tobacco smokers ranged from 17 years to 24.7 years, and for the control population their mean age ranged from 16.6 years to 23.3 years (30, 44, 55). Most of the studies were conducted on a predominantly male population except for two studies that had more females than males (30, 53). The average amount of time in education ranged from 10.1 years to 13.8 years. However, of the included studies that were pooled, several did not include data for the years of education (44, 53–55). Most of the papers included in this meta-analysis reported pack-years, years of smoking, and cigarettes per day, except for one study that did not report any of these data (55) and two studies not mentioning pack-years specifically (30, 54). Pack-years ranged from 3.5 to 6.4 (18, 43, 44), years of smoking ranged from 2 years to 7.3 years (44, 54), and cigarettes per day ranged from 11.7 Jacobsen et al. (30) cigarettes per day to 16.9 cigarettes per day (21) (**Table 1**).

**Table 1:** Demographics and smoking characteristics ^♦^.

Authors and year of publication	Quality of studies	Country	Study Type	Smokers Number (N)	Age (Mean ± SD) in years	Sex	Years of Education (± SD)	Pack-years	Years of smoking	Cigarettes per day	Poly-substance use	Non-smokers Number (N)	Age (Mean ± SD) in years	Sex	Years of Education (± SD)	Poly-substance use
Maurage et al. (53)	Good	Belgium	Case-Control	25	21.4± 2.3	10 Males	NA	4.2± 4.3	6.0 ± 3.5	13.1 ± 4.4	None	25	22.4± 2.3	8 Males	NA	None
Al-Mshari et al. (44)	Good	Saudi Arabia	Case-Control	31	24.7 ± 4.1	Males	NA	6.4 ± 8.3	7.3 ± 5.0	14.8 ± 9.2	None	42	20.9± 2.7	Males	NA	None
Bashir et al. (55)	Fair	Saudi Arabia	Case-Control	22	24.4± 5.3	Males	NA	NA	NA	NA	None	30	23.3± 2.7	Males	NA	None
Li et al. (5)	Fair	China	Case-Control	31	19.4 ± 1.3	Males	12.5 ± 0.7	3.7 ± 1.2	4.9 ± 1.9	15.0 ± 7.1	None	30	19.5± 1.5	Males	12.7± 0.7	None
Bi et al. (43)	Fair	China	Case-Control	40	19.6 ± 1.9	Males	12.0 ± 1.3	3.5 ± 2.4	4.2 ± 1.9	15.6 ± 5.3	None	40	19.8± 2	Males	12.2 ± 1.5	None
Yuan et al. (21)	Fair	China	Case-Control	60	20.0 ± 1.7	53 Males	13.8 ± 0.7	3.6 ± 1.7	4.4 ± 1.6	16.9 ± 5.4	None	60	19.9± 1.8	52 Males	13.6 ± 0.9	None
Zhao et al. (54)	Fair	China	Case-Control	30	21.4 ± 2.1	NA	NA	NA	≥ 2	16.8 ± 2.51	None	31	21.1± 1.2	NA	NA	None
Feng et al. (18)	Fair	China	Case-Control	27	20.7 ± 1.5	Males	12.6 ± 0.9	3.5 ± 2.4	4.9 ± 2.2	14.1 ± 4.6	None	25	20.5± 1.4	Males	12.6 ± 0.8	None
Jacobsen et al. (30)	Good	United States	Case-Control	41	17.0 ± 1.1	14 Males	10.1 ± 1.1	NA	3.9 ± 0.7	11.7 ± 6.7	None	32	16.6± 1.3	12 Males	10.1 ± 1.4	None

N, total number in study; Pack-Years, a person’s cigarette consumption calculated as the packs of cigarettes smoked per day, multiplied by the length of consumption in years; NA, Not Available; SD, Standard Deviation.

♦ Note. One of the studies did not provide the gender of the population used in their study. These data are shown in the above table as they were provided in the respective studies.

### 3.3 Neurocognitive tests

In the papers that were pooled for this systematic review, researchers used specific neurocognitive tests to investigate the neurocognitive domains of adolescent and young adult chronic tobacco smokers (**Table 2**). The Stroop colour-word task, used to test motor impulsivity and cognitive flexibility, was the most utilised neurocognitive test in the studies included in this review (5, 18, 21, 43). The Wechsler Adult Intelligence Scale Third Edition (WAIS-III) was used by four of the included studies to specifically measure intelligence in chronic tobacco smoking adolescents and young adults (18, 21, 30). As well as using WAIS-III, the domain of attention was measured using several tests throughout the papers included. One of the studies, by Bashir et al. (55), assessed attention in chronic tobacco smoking adolescents and young adults using the Attention Switching Task (AST). This study included another neurocognitive domain, Pattern Recognition Memory Task (PRM), to measure learning and memory (both short- and long-term memory) in the chronic tobacco smoking group. Maurage et al. (53) assessed attention in chronic tobacco smoking cohort with the Attention Network Test (ANT). Li et al. (5) also explored how attention is affected by adolescent and young adulthood chronic tobacco smoking by measuring Reaction Time (RT). Al-Mshari et al. (44) used numerous tests to assess multiple neurocognitive domains. This included RT to test for attention, as well as the Spatial Working Memory Task (SWM) to investigate any association between impairments in spatial working memory and chronic tobacco smoking status in adolescents and young adults. The same study also used the Multitasking test (MTT) and Rapid Visual Information Processing Task (RVIP) to assess the participants’ attention and impulsivity. Zhao et al. (54) used the Go/No-Go Task to assess motor impulsivity in their assessments of chronic tobacco smokers. Finally, as well as exploring intelligence, Jacobsen et al. (30) included four different neurocognitive tests: the Hopkin’s Verbal Learning Test (HVLT) was used to assess verbal learning and memory; the Auditory n-Back Task was used to assess working memory; the Kauffman Brief Intelligence Test (KBIT) was used to assess intelligence; and the Continuous Performance Test (CPT) was used to assess selective, divided, and sustained attention in chronic tobacco smokers.

**Table 2:** Neurocognitive tests and domains.

	Neurocognitive Tests	Neurocognitive domains
Maurage et al. (53)	ANT	Attention
Al-Mshari et al. (44)	RT SWM MTT RVIP	Attention, Spatial Working Memory, and Impulsivity
Bashir et al. (55)	AST PRM	Attention & Learning and Memory (Short-Term and Long-Term memory)
Li et al. (5)	Stroop colour-word task RT	Cognitive Flexibility & Attention
Bi et al. (43)	Stroop colour-word task	Cognitive Flexibility
Yuan et al. (21)	Stroop colour-word task	Cognitive Flexibility
Zhao et al. (54)	Go/no-go task	Motor Impulsivity
Feng et al. (18)	Stroop colour-word task	Cognitive Flexibility
Jacobsen et al. (30)	HVLT KBIT Auditory n-back task CPT	Verbal Learning and Memory, Intelligence, Working Memory, and Selective, Divided & Sustained Attention.

AST, Attention Switching Task; SOC, Stockings of Cambridge; PRM, Pattern Recognition Memory; CRT/DR2, Sample Choice Reaction Time; RT, Reaction Time; RVP, Rapid Visual Information Processing; SWM, Spatial Working Memory; SST, Stop Signal Test; CGT, Cambridge Gambling Task; WAIS-III, Wechsler Adult Intelligence Scale/Third Edition; TVPS, Test of Visual Perceptual Skills; KBIT, Kauffman Brief Intelligence test.

### 3.4 Qualitative analysis

All 9 studies were included in this qualitative systematic review. The 9 selected papers assess neurocognitive functional impairments associated with chronic tobacco smoking in adolescence and young adulthood (5, 18, 21, 30, 43, 44, 53–55).

Maurage et al. (53) proposed that when looking into attention (alerting, orienting and executive control) using the Attention Network Test (ANT), adolescent chronic tobacco smokers face more impairments in executive control when compared to non-smokers of the same age range. Additionally, they reported adolescent and young adult chronic tobacco smokers show impairments in attention and reaction time compared to healthy non-smoking individuals of the same age group (53), having slower reaction times in incongruent stimuli and having difficulty ignoring the distractors used. They have also suggested that there is a significant association between the chronic smoking group and negative urgency (p<0.05), positive urgency (p=0.01) and lack of premeditation (p=0.02), where they had higher scores than the healthy control group.

Al-Mshari et al. (44) provided evidence that adolescent and young adult chronic tobacco smokers show higher levels of cognitive impairments in comparison to non-smokers of the same age group. These cognitive impairments were in sustained attention, as assessed by the Rapid Visual Information Processing task (RVIP), and attention and impulsivity, as assessed by the Multi-Tasking Tests (MTT). This study additionally showed that there is a significant difference in Rapid Visual Information Processing A (RVPA) (p=0.001), Rapid Visual Information Processing Probability of False Alarm (RVPPFA) (p=0.027), and Multi-Tasking Test Reaction Latency (Median) (MTTLMD) (p=0.007) in the performance of non-smokers when compared to smokers, with non-smokers performing much better in these domains. Insignificant differences (p>0.05) between young adult smokers and non-smokers were reported in relation to spatial working memory as assessed by the Spatial Working Memory Strategy (SWMS) test. Furthermore, the researchers propose that the occurrence of impairments in sustained attention and executive function in young smokers is supported by previous studies that provide the same results (56–58).

Bashir et al. (55) proposed that chronic tobacco smokers exhibited notable deficits in neurocognitive function, as demonstrated by the Attention-Switching Task (AST) and Pattern Recognition Memory (PRM) tests. The AST test revealed that chronic tobacco smokers had significantly higher values in the AST-latency (p=0.001), congruent (p=0.001) and incongruent (p=0.001) conditions compared to non-smokers, indicating impaired attention, memory, and reaction time tasks between the two groups. Although the PRM test was also utilised, no significant difference (p=0.101) was found between the two groups. This would suggest that adolescent or young adult chronic tobacco smokers have a significant difference in their performance when compared to non-smokers in reaction time and attention. They also suggest that the lack of difference in the performances of both groups in the PRM test can be due to memory function preservation in smokers.

According to Zhao et al. (54), the authors observed that in a specific stimulus in the go/no-go task (600 ms), there was no significant (p>0.05) difference between the chronic tobacco smoking group compared to the non-smoking controls. However, when changing the stimulus of the test (200 ms), the smoking group had more significant (p<0.001) on-the-go and no-go phases when compared to the non-smoking controls. Additionally, when using the short stimulus on the go task, the chronic smoker group had a much faster response to the stimulus (RT) when compared to the non-smoking control. This describes how chronic tobacco smoking may not only be associated with impairments in the go/no-go tasks, but that chronic smokers also tend to increase their reaction time in tasks that involve responding to a fast stimulus and, additionally, make more errors.

Feng et al. (18) used the colour-word Stroop Task to measure response errors, reaction times, and response delays of participants under congruent and incongruent conditions. The results showed that smokers made a significantly higher number of incongruent errors (p<0.05) and had significantly shorter reaction delay times (p<0.05) compared to non-smokers. The results also provided evidence of minor, but non-significant, differences in the other conditions including congruent errors, and incongruent and congruent reaction times. These findings were further supported by Bi et al. (43), who also used the colour-word Stroop Task and found that smokers had a longer reaction time during congruent conditions (p<0.001) compared to non-smokers. The smoking group also showed a significant difference in their scores in reaction delay (p<0.05), where they had shorter reaction delay scores when compared to the non-smoker group. Additionally, the smoker group made more errors during the incongruent condition (p<0.01) compared to the non-smoker group, which was also observed in the study by Yuan et al. (21) in the colour-word Stroop Task, where they also provided evidence that smokers had significantly more errors (p<0.05) and shorter reaction delay times (p=0.005) in the incongruent condition. Similarly, Li et al. (5), using the colour-word Stroop Task, found that both tobacco smokers and non-smokers made more errors (p<0.005) and had shorter reaction delay times (p<0.01) during the incongruent condition compared to the congruent condition. They all noted a trend of shorter response delay in adolescent and young adult smokers when compared to non-smokers (5, 18, 21, 43). Additionally, it was reported by Li et al. (5), (p<0.001), Bi et al. (43) (p<0.001), and Yuan et al. (21) (p<0.005) that there was a significant Stroop effect noticed in both smoking and non-smoking groups, where they demonstrated longer reaction times when performing in the incongruent conditions compared to when performing in the congruent condition. Studies by Feng et al. (18), and Yuan et al. (21) also performed WAIS III on adolescent and young adult chronic tobacco smokers in order to measure their intelligence quotient (IQ), where they proposed that there were no significant differences between the adolescent or young adult smoking group and the non-smoking group.

Jacobsen et al. (30) reported adolescent smokers perform significantly (p<0.05) less accurately on the dichotic 1-back and 2-back conditions, and the binaural 1-back condition of an n-back task in comparison to non-smokers. It proposes that adolescent smokers have impairments in working memory in comparison to adolescent non-smokers. These impairments were found to be more severe during a nicotine withdrawal condition. No significant differences (p>0.05) were identified between the two groups in relation to verbal memory during a nicotine-satiated condition. However, verbal memory, as assessed by the Verbal Learning Test-Revised (HVLT-R), worsened during nicotine withdrawal for adolescent smokers. No significant differences (p>0.05) were detected in relation to sustained, selective, and divided attention task performance accuracy between adolescent smokers and non-smokers. Nonetheless, as stated by the same authors “across test sessions, smokers performed this attention task significantly more slowly than did non-smokers [smokers reaction time (RT) = 1056.7 ± 305.9 msec, non-smokers RT = 944.5 ± 262.5 msec; β = 144.0, t (65) = 2.1, p = .04]” (30). Group differences in reaction time did not vary between nicotine-satiated and withdrawal conditions.

There were variations in the nicotine administration state of chronic smokers between the studies. Two studies did not mention the duration since the last nicotine administration at all, reflecting a less detailed assessment of participants (44, 55). Two studies assessed participants at 30 minutes post-administration (18, 43). While this is soon after administration, the studies demonstrated statistically significant impairments in smokers versus non-smokers in multiple neurocognitive tests. Three studies assessed smokers at 1 hour after administration (5, 21, 54). Li et al. (5) elaborated on this, stating that no chronic smoking participants demonstrated an urge to smoke during the pre-testing questionnaire. Maurage et al. (53) alone measured smokers at 3 hours post-administration. This study found that the executive function of heavy smokers was independent of current tobacco craving, as measured in the pre-test questionnaires, and correlated more with the heaviness of smoking rather than the duration of smoking. Jacobsen et al. (30) most comprehensively assessed the effects of nicotine withdrawal on neurocognitive performance. Their chronic smoking participants were assessed twice: the first was following “ad libitum” smoking (smoking as one wishes – no clear definition), and then again two weeks later at 24 hours post-administration of nicotine. From this, Jacobsen et al. (30) suggested an association between nicotine withdrawal (at 24 hours) and the domains of working memory and short-term verbal memory, with no associations determined elsewhere.

### 3.5 Quantitative analysis

#### 3.5.1 Motor impulsivity

**Figure 2:**
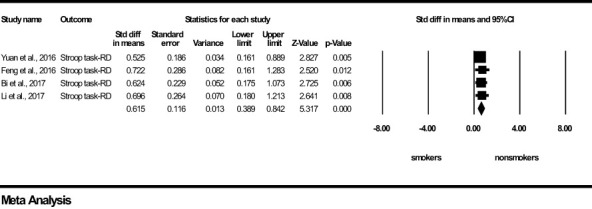
Motor impulsivity-Stroop Task-Reaction Delay-forest plot (std diff, standard difference; Z value, one sample z statistics; p value, probability that Z statistics is significantly different than 0; Lower limit, lower limit of the 95% confidence interval for the effect size; Upper limit, upper limit of the 95% confidence interval for the effect size; RD, Reaction delay).

For *Motor-Impulsivity*-Stroop Task-Reaction Delay, a significant and medium effect size was found in favour of the tobacco non-smoker group (z=5.317, *p*<0.0001), indicating that young non-smokers take more time/are less impulsive when reacting between congruent and incongruent conditions compared to young chronic tobacco smokers (**Figure 2**). Results of *Q* and I^2^ tests indicated the absence of heterogeneity between the pooled studies (*Q*=0.471, *p*=0.925, I^2 ^= 0.000). Visual inspection of Funnel’s Plot revealed the absence of publication bias (**Supplementary Figure 1**).

**Figure 3:**
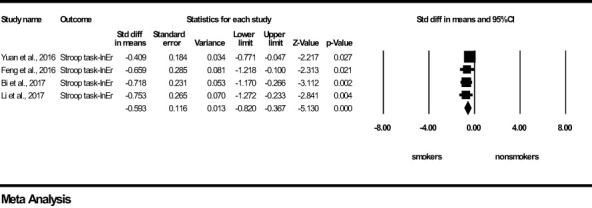
Motor impulsivity-Stroop Task-Incongruent Errors-forest plot (std diff, standard difference; Z value, one sample z statistics; p value, probability that Z statistics is significantly different than 0; Lower limit, lower limit of the 95% confidence interval for the effect size; Upper limit, upper limit of the 95% confidence interval for the effect size; InEr, Incongruent errors).

For *Motor-Impulsivity*-Stroop Task-Incongruent Errors, a significant and medium effect size was found in favour of the young chronic smoker group (z=-5.130, *p <*0.0001), indicating that young chronic tobacco smokers make more errors during cognitive conflict conditions compared to young non-smokers (**Figure 3**). Results of *Q* and I^2^ tests indicated the absence of heterogeneity between the pooled studies (*Q*=1.705, *p*=0.636, I^2^ = 0.000). Visual inspection of Funnel’s Plot revealed the absence of publication bias (**Supplementary Figure 2**).

#### 3.5.2 Intelligence

**Figure 4:**
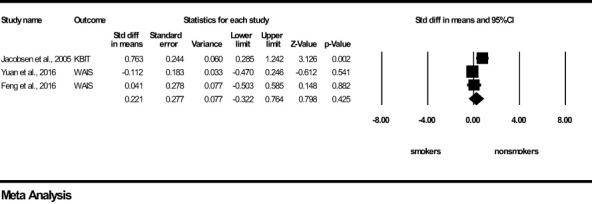
Intelligence forest plot (std diff, standard difference; Z value, one sample z statistics; p value, probability that Z statistics is significantly different than 0; Lower limit, lower limit of the 95% confidence interval for the effect size; Upper limit, upper limit of the 95% confidence interval for the effect size; WAIS, Wechsler Adult Intelligence Scale; KBIT, Kauffman Brief Intelligence test).

For *Intelligence*, a non-significant and small effect size was found in favour of the young non-smoker group (z=0.798, *p*=0.425) (**Figure 4**). Results of *Q* and I^2^ tests indicated heterogeneity between the pooled studies (*Q*=8.496, *p*<0.05, I^2^ = 76.459). Visual inspection of the Funnel’s Plot revealed moderate publication bias (**Supplementary Figure 3**).

#### 3.5.3 Working memory

**Figure 5:**
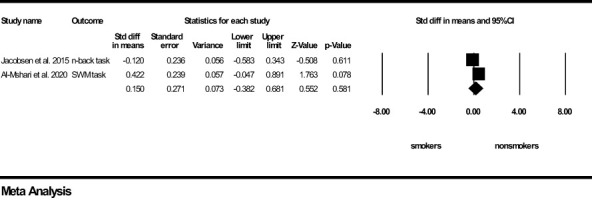
Working Memory forest plot (std diff, standard difference; Z value, one sample z statistics; p value, probability that Z statistics is significantly different than 0; Lower limit, lower limit of the 95% confidence interval for the effect size; Upper limit, upper limit of the 95% confidence interval for the effect size; SWM, Spatial Working Memory).

For *working memory*, a non-significant and small effect size was found in favour of the young non-smoker group (z=0.150, *p*=0.581) (**Figure 5**). Results of *Q* and I^2^ tests indicated small heterogeneity between the pooled studies (*Q*=2.599, *p*=0.107, I^2^ = 61.517). It was not possible to compute a Funnel’s plot to assess publication bias as the number of included studies was too low.

## 4 Discussion

### 4.1 Summary of results and key findings

This systematic review and meta-analysis were conducted to provide a quantitative synthesis regarding the association between chronic tobacco smoking and neurocognitive impairments during adolescence and young adulthood (**Table 3**). Both quantitative and qualitative analysis results showed an association between chronic tobacco smoking and impaired motor impulsivity in chronic tobacco smoking adolescents and young adults, while qualitative analysis of these nine studies demonstrated that smoking behaviours in younger age groups may be associated with impairments of various neurocognitive domains. Of the papers included in this study, Li et al. (5) Yuan et al. (21) Bi et al. (43), and Feng et al. (18) propose that early onset of tobacco smoking is associated with neurocognitive impairments in the domain of attention, specifically when performing incongruent error and reaction delay tasks. Jacobsen et al. (30) provided evidence that adolescent smoking may be associated with impairments in working memory. Al-Mshari et al. (44) Maurage et al. (, 53), and Bashir et al. (55) suggest that adolescent and young adulthood smoking can be associated with impairments in executive function and attentional domains. Finally, Zhao et al. (54) have provided evidence that there are associations between adolescent or young adulthood smoking and impairments in the domains of intelligence and impulsivity respectively.

**Table 3:** Compiled effect sizes for each neurocognitive domain.

		Effect size and 95% CI				Test for Null (2 fail)		Heterogeneity		
Neurocognitive Domain	Studies	Effect Size	SE	Lower Limit	Upper Limit	Z	p for Z	Q	p for Q	I^2^
Motor Impulsivity										
*a. Reaction delay in ST*	4	0.615	0.116	0.389	0.842	5.317	0.000**	0.471	0.925	0.000
*b. Incongruent errors in ST*	4	-0.593	0.116	-0.820	-0.367	-5.130	0.000**	1.705	0.636	0.000
Intelligence										
*Intelligence*	3	0.221	0.277	-0.322	0.764	0.798	0.425	8.496	<0.05	76.459
Working Memory										
*Working Memory*	2	0.150	0.271	-0.382	0.681	0.552	0.581	2.599	0.107	61.517

N, Total number of studies. P, Significance, * significant at the p < 0.05 level. ** significant at the p < 0.01 level. CI , Confidence interval; SE, Standard error; Z value, One sample z statistics; p value, Probability that Z statistics is significantly different than 0; Q, Cochran’s Q; I2, Percentage of variance due to heterogeneity.

The association between chronic tobacco smoking and these neurocognitive impairments support the findings of the literature review conducted by Campos et al. (59) and of the systematic review and meta-analysis conducted by Conti et al. (39). However, the findings of the systematic review and meta-analysis conducted by Conti et al. (39) are primarily related to middle-aged adult chronic tobacco smokers, unlike the population used in the current study. Therefore, the findings of this review and meta-analysis contribute to the body of literature by showing an association between chronic tobacco smoking and neurocognitive impairments in younger age groups with a shorter smoking history.

### 4.2 Mechanistic interpretations

In the context of neurocognition, exposure to nicotine, either directly or indirectly, has been associated with neurobiological changes (21, 39, 60, 61). According to Conti et al. (39), exposure to nicotine is linked to complex cognitive modulation, where acute nicotine use may enhance cognitive functions, particularly in the domains of attention and memory (39, 62, 63). Aside from changes to working memory and attention, chronic nicotine exposure may be associated with neurocognitive impairments in impulse control, the speed of processing information, intellectual ability, auditory-verbal memory, and vocabulary (oral arithmetic, receptive and expressive) (4, 30, 64–66). In the qualitative analysis of Maurage et al. (53), their data suggests that young chronic tobacco smokers have more difficulty inhibiting or resisting irrelevant or distracting stimuli when focusing on relevant ones. Additionally, they indicate that chronic smokers have deficits in impulsivity and executive attentional control.

One mechanistic interpretation for the findings of this study could consist of the neurotoxic effect of nicotine on the developing adolescent brain as proposed by the Tobacco-Induced Neurotoxicity theory of Adolescent Cognitive Development (TINACD) (67). According to this paradigm, chronic tobacco smoking at younger ages may lead to structural and functional impairments in frontostriatal brain regions (e.g. PFC, ACC) modulating cognitive control, attention, and impulsivity. Alongside this, a neuroimaging study conducted by Conti and Baldacchino (68) reported a correlation between the age of regular smoking initiation during adolescence (16 years) and reduced Gray Matter (GM) volume in the VLPFC of chronic tobacco smokers (69, 70). Nicotine exposure is also associated with accelerated brain ageing and brain structural damage through its neurotoxic properties, which in turn may be associated with the reinforcing and inducing of other forms of substance dependencies (70).

The results of this meta-analysis will need to consider likely confounders. One significant consideration is that these young individuals may demonstrate neurocognitive impairments, such as impulsivity, prior to initiating smoking, meaning that the neurocognitive phenotypes demonstrated an increase in the probability of an adolescent or a young adult picking up the tobacco smoking behaviour. This is likely to be attributable to complex socioeconomic factors involved, such as education, adverse childhood experiences and other social disadvantages (71). Subsequent negative effects or neurocognitive impairments caused by chronic tobacco smoking may then lead to further impulsive reactions to avoid the unwanted negative effects of tobacco smoking cessation, causing these adolescents and young adults to continue this smoking behaviour, establishing a positive feedback loop (72). Determining the relationship between socioeconomic factors and an individual’s neurocognitive outcome in chronic adolescent smokers is of high importance.

Impulsivity has been shown to be a primary reason for the initiation of tobacco smoking, as well as the sustainment of this habit to help avoid the aversive and negative consequences of abstinence from smoking (73). According to Balevich et al. (74), it is hypothesized that this impulsiveness to initiate smoking is related to sensation seeking (reward-seeking) and curiosity while the impulsiveness to sustain the smoking behaviour is related to disinhibitory impulsiveness. This disinhibitory impulsiveness is related to the aversion of the negative effects of cessation, which are associated with nicotine dependence. As chronic tobacco smokers are at risk of dependence, this is a form of impulsiveness that is of high importance (75). Young adults who are chronic smokers also show more risk-taking behaviours than their counterparts (4, 76).

Intriguingly, when considering the younger ages of the individuals included in this meta-analysis, it is proposed that neurocognitive impairments are associated with a relatively short history of chronic tobacco smoking. This may suggest that smoking at an early stage of life predisposes the brain to progressive neurocognitive impairments, (e.g. heightened motor impulsivity). This may lead to the development of compulsive tobacco-seeking and smoking behaviour during adulthood, therefore negatively impacting quality of life and increasing the risk of adverse health outcomes (77). This proposed relationship between early onset tobacco smoking and compulsive tobacco smoking during adulthood, however, remains speculative at this stage due to the lack of robust longitudinal studies.

### 4.3 Strengths and limitations of the methods used and the results

To gather both qualitative and quantitative data, various online databases were used to identify the studies pooled for this systematic review and meta-analysis. The inclusion and exclusion criteria were stringent and allowed us to exclude participants with concurrent psychiatric illness, excessive alcohol intake, or polydrug use, as these were considered confounding variables.

The number of papers used in the current meta-analysis was low due to the lack of relevant research conducted on adolescents and young adults. This may have affected the results of the meta-analysis testing the association between chronic tobacco smoking and working memory impairments, as it was only possible to include three studies. There are discrepancies between the results of the quantitative and qualitative analyses for the domains assessed. This may have occurred due to the low number of studies that could be included in the meta-analysis and may also be the result of studies utilising different neurocognitive tests to assess the neurocognitive domains.

The reliability of results may be affected by including case-control studies, which are considered non-randomised studies (NRS). This may allow a larger or more unpredictable uncalculated bias to cause an underestimation or overestimation of the results (78). The inclusion of case-control NRSs is due to the lack of Randomized Controlled Trials (RCTs) carried out on chronic tobacco smoking.

The results of the meta-analysis identified an association between adolescent and young adult chronic tobacco smoking and neurocognitive impairments from cross-sectional data. Therefore, a direct causation cannot be inferred. Many other substances, such as alcohol, opioids, and stimulants, have been extensively explored in individuals, and subsequently have shown to affect neurocognitive functions (42, 79–81). The results of this meta-analysis could be considered confounders for these individuals, as the users of these substances are likely to be concurrent chronic tobacco smokers, which may account for a degree of neurocognitive impairment identified in users of other substances (39, 82–84). In the studies included in the current systematic review and meta-analysis, the number of pack years was not consistent and not reported in some studies. This may be also considered a confounding factor as research has shown a negative association between neurocognitive impairments and the number of pack-years (39).

The results of the study may have been influenced by confounding variables. This includes sociodemographic factors such as socioeconomic status, level of education, adverse childhood experiences, and parental difficulties. These variables have been shown to negatively affect the neurocognitive abilities of individuals (51, 85–90).

Considering that the neurocognitive impairments identified by the current review may have been pre-morbid, longitudinal studies would be needed to investigate the directionality of the association between chronic tobacco smoking and neurocognitive impairments in adolescents and young adults. One such example has been demonstrated in a longitudinal study using Scottish data, where a lower childhood intelligence was found to be associated with a higher risk of becoming a smoker and continuing to smoke throughout life (91). Another confounding factor is the prevalence of concurrent undiagnosed neurodevelopmental disorders, such as attention deficit hyperactivity disorder (ADHD), in the assessed populations, which may influence the measured outcomes in neurocognitive testing.

A limitation of the studies being analysed during systematic review and meta-analysis is the lack of consistency in the nicotine withdrawal state of the tested chronic tobacco smoking participants. This is demonstrated through the insufficient descriptions of nicotine states and the variability in post-administration durations prior to assessment. Two studies did not include any information on this at all. This variability impacts the direct comparison of results between studies. It is important as the effects of nicotine withdrawal can begin after 4 hours, up until 3 days from the last administration of the nicotine (92). Using this cut-off, all the studies that declared the duration between the last administration of nicotine and neurocognitive assessments are within a defined and comparable period of nicotine administration that excludes states of nicotine withdrawal (5, 18, 21, 30, 43, 53, 54). Additionally, the statistically significant impairments in smokers versus non-smokers in multiple neurocognitive tests conducted shortly after nicotine administration suggest that any acute neurocognitive enhancing effects of nicotine administration were limited.

### 4.4 Clinical relevance

The neurocognitive impairments identified by the current review could be targeted by therapies such as Cognitive Rehabilitation Treatments (CRTs) and pre-treatment neuropsychological assessments, as aids for smoking cessation programs. CRTs are specialised procedures used to treat or improve neurocognitive functions, such as attention, problem-solving, learning and memory, and planning (39). Adolescents and young adults who are chronic smokers show more impulsivity in their decisions than their counterparts, therefore, some treatments that target this neurocognitive domain, such as Dialectical Behavioural Therapy (DBT) or Cognitive Behavioural Therapy (CBT), may be beneficial in smoking cessation programs (39, 93–96).

Components of psychological therapy will benefit from improving the understanding of neurocognitive associations with chronic tobacco smoking, and other substance abuse disorders. Psychoeducation (PE) typically involves educating a patient about their condition to explore the emotional and motivational components they experience, which aims to improve the efficacy of treatment for that individual (97). This can be combined with education on related neuroscientific pathophysiology of a health condition, termed neuroscience-informed psychoeducation (NIPE). This could include any neurocognitive associations of chronic tobacco smoking, which the healthcare professional can employ to provide the patient with an enhanced understanding, and therefore improve their insight and decision-making, whilst also destigmatising the challenges of the conditions, leading to better compliance with treatment (97). An example of the application of neuroscientific understanding to PE is the program termed “Neurocognitive Empowerment for Addiction Treatment” (NEAT), which is planned to be implemented on patients with substance abuse disorders in an RCT undertaken by Ekhtiari et al. (98).

The socioeconomic associations with adverse long-term health and social outcomes have become well established, as described in the WHO Commission on Social Determinants of Health in 2008, as well as many other government-affiliated and independent institutions (99–101). By understanding the interactions between the determinants of health and the specific outcomes in adolescent chronic tobacco smokers, policymakers can target appropriate interventions. For example, recent data from the UK show that one in four unemployed adults are smokers, almost twice the probability of an employed person, and over 28% of people with no formal qualifications are smokers, compared to 12% of people who have obtained higher education (8). Using data from the English Index of Multiple Deprivation, the rate of smoking in the population (over the age of 16) is 19% for the most deprived quintile, in comparison to 6% for the least deprived quintile (6). Identifying adolescent smokers as a high-risk group thus allows policymakers to target the population group’s circumstances that contribute most to the increased risk of commencing smoking during adolescence, such as school attendance or adverse childhood experiences, as well as their carers’ social circumstances including education and social capital, to improve overall long-term health and social outcomes across the population (71, 100, 102–104). Additionally, the syndemic nature of poor socioeconomic factors compounded by cognitive impairments at an early stage of tobacco smoking will be associated with a reduced probability of quitting smoking as an adult (105).

The significant increase in the adoption of electronic nicotine delivery systems (ENDS, also known as vapes or e-cigarettes) will be an ongoing concern. Survey data from the Action on Smoking and Health (ASH), carried out on the UK population, show that in 2022 more young people (11-18 years old) had participated in the use of vaping products (8.6%) than tobacco smoking (6.0%), in comparison to previous years, in which tobacco smoking had been more prevalent (106). A recent paper investigating ENDS by Wade et al. (107) suggests that the use of ENDS in 16-22-year-old participants is not associated with any neurocognitive impairments, once controlled for alcohol use, substance use, and sociodemographic factors. It demonstrated that there were no significant differences in neurocognitive performance between nicotine users and nicotine-naïve users, whilst the comparison between the ENDS and the tobacco-smoking group is confounded by the concurrent use of ENDS by the tobacco-smoking group.

## 5 Conclusion

This systematic review and meta-analysis proposed a cross-sectional relationship between chronic tobacco smoking and neurocognitive impairments in adolescents and young adults. The number of studies pooled for both qualitative and quantitative analyses was, however, relatively low, suggesting that further research is needed to investigate the cross-sectional relationship between chronic smoking and neurocognitive impairments in young people. Furthermore, longitudinal studies are needed to investigate the temporal relationship between tobacco smoking uptake during adolescence or young adulthood and neurocognitive impairments. A comprehensive understanding of the relationship between young smokers and adverse neurocognitive outcomes may provide opportunities to optimise clinical and public health policymaking to improve outcomes in mortality, morbidity, and quality of life. This is especially important in the context of the increasing popularity of alternative methods of nicotine administration, such as e-cigarettes or vapes, which also need particular focus.

## Data availability statement

The original contributions presented in the study are included in the article/**Supplementary Material**. Further inquiries can be directed to the corresponding author.

## Author contributions

AE: Data curation, Formal analysis, Investigation, Methodology, Software, Visualization, Writing – original draft, Writing – review & editing. SV: Data curation, Formal analysis, Investigation, Methodology, Software, Writing – original draft, Writing – review & editing. AC: Conceptualization, Data curation, Formal analysis, Methodology, Software, Supervision, Visualization, Writing – original draft, Writing – review & editing. AB: Conceptualization, Funding acquisition, Methodology, Project administration, Supervision, Writing – review & editing.

## Supplementary material

Supplementary Figure 1 Funnel’s plot for Motor impulsivity-Stroop Task-Reaction Delay.

Supplementary Figure 2 Funnel’s plot for Motor impulsivity-Stroop Task-Incongruent errors.

Supplementary Figure 3 Funnel’s plot for Intelligence.

Supplementary Table 1 Impulsivity.

Supplementary Table 2 Cognitive Flexibility and Attention.

Supplementary Table 3 Memory and learning.

Supplementary Table 4 National Institutes of Health Study Quality Assessment Questionnaire.

Supplementary Table 5 PRISMA Checklist.
