# Supplementary figures and images for: Chronic tobacco smoking and neurocognitive impairments in adolescents and young adults: a systematic review and meta-analysis
Source: Front Psychiatry. 2024 Apr 23;15:1384408. doi: 10.3389/fpsyt.2024.1384408 (PMC11074441; doi:10.3389/fpsyt.2024.1384408)

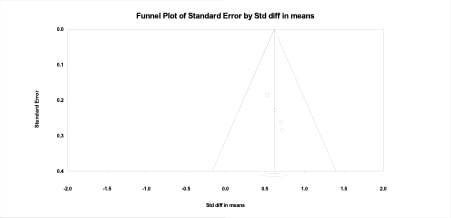

Supplement: Supplementary Figure 1 — Funnel’s plot for Motor impulsivity-Stroop Task-Reaction Delay. [file Image_1.jpg]

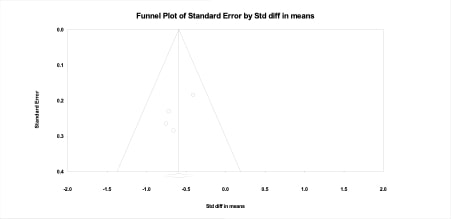

Supplement: Supplementary Figure 2 — Funnel’s plot for Motor impulsivity-Stroop Task-Incongruent errors. [file Image_2.jpg]

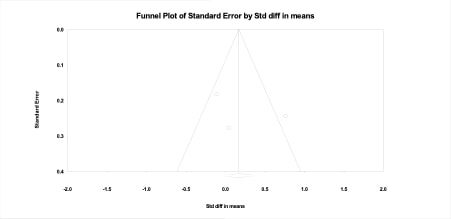

Supplement: Supplementary Figure 3 — Funnel’s plot for Intelligence. [file Image_3.jpg]
