# Supplementary material for: Chronic tobacco smoking and neurocognitive impairments in adolescents and young adults: a systematic review and meta-analysis
Source: Front Psychiatry. 2024 Apr 23;15:1384408. doi: 10.3389/fpsyt.2024.1384408 (PMC11074441; doi:10.3389/fpsyt.2024.1384408)
Supplement: Supplementary Table 1 — Impulsivity. [file DataSheet_1.docx]

**Supplementary Table 1:** *Impulsivity*

| **Main Domain** | **Subdomain** | **Alternative Name** | **Definition** | **Tests** |
| --- | --- | --- | --- | --- |
| ***Cognitive Impulsivity*** |  | Delay discounting or urgency | Ability to opt for larger delayed rewards over smaller more immediate rewards |  |
|  | 1. Reflection Impulsivity |  | Decision-making under ambiguity | IGT, MFFT, BIS, DDT |
|  | 1. Risk Taking |  | Decision-making under risk | CGT, IGT, RDMT, GDT |
| ***Motor Impulsivity*** |  | Inhibitory control | Ability to suppress emotional, cognitive, and behavioural responses |  |
|  | 1. Behavioural inhibition | Motor response inhibition | Process required to stop a planned movement | AGN, SS, Go/NoGo |
|  | 1. Cognitive inhibition | Focused Attention | Process required to suppress a salient but conflicting stimulus while identifying less salient ones | ST |
| ***Non-planning impulsivity*** | Reasoning and problem solving | Central executive in working memory model Lack of premeditation | Ability to think ahead and actively search for an appropriate solution | TOL, SOC, ROCFT, PMT, TOH WAIS-III (Block Design, Matrix Reasoning), SS, SWM |

IGT = Iowa Gambling Task; SWM = Spatial Working Memory; MFFT = Matching Familiar Figures Task; BIS = Barratt Impulsiveness Scale; DDT = Delay Discounting Test; RDMT = Rodgers Decision Making Task; CGT = Cambridge Gambling Task; GDT = Game and Dice Test; AGN = Affective Go-NoGo; SS = Spatial Span; ST = Stroop Test; TOL = Tower of London; SOC = Stockings of Cambridge; ROCFT = Rey-Osterreith Complex Figure Test; PMT = Proteus Maze Test; WAIS-III = Wechsler Adult Intelligence Scale-Third Edition.

**Supplementary Table 2:** *Cognitive Flexibility and Attention*

| **Main Domain** | **Subdomains** | **Alternative Names** | **Definition** | **Tests** |
| --- | --- | --- | --- | --- |
| ***Cognitive Flexibility*** |  | Rigidity | Ability to shift avenues of thought and action in order to perceive process and respond to situations in different ways |  |
|  | 1. Reactive flexibility | Perseveration or shifting of perceptual set | Ability to realign a behavioural predisposition to altered contingencies | WCST, ST, IED, TMT, SCT, MCST |
|  | 1. Spontaneous flexibility or fluency | Verbal and non-verbal fluency | Requires the intrinsic generation of responses or alternatives | COWAT, FAS, VFT, RFFT, WAIS III (Similarities), RWT, DF |
| ***Attention*** | 1. Deployment | 1. Arousal |  | Observation |
|  |  | 1. Focused and selected attention | Ability to reject irrelevant information while attending to relevant input | WAIS-III (Digit Span), TMT, TEA, ST, AGN |
|  |  | 1. Sustained attention | Readiness to detect rarely and unpredictable occurring signals over prolonged periods of time | PASAT, TOVA, TEA, CFT |
|  | 1. Capacity/encoding or data processing |  | Ability for individuals to hold information in mind and process OR need to process tasks simultaneously |  |
|  |  | 1. Attention Span |  | CVLT, RAVLT |
|  |  | 1. Recreation time or information processing speed |  | DSST, WAIS (Digit Symbol) |

WCST = Wisconsin Card Scoring Test; ST = Stroop Test; IED = Intra/Extra-Dimensional Set Shifting Task; TMT = Trail Making Test; SCT = Logan Stop Change Task; MCST = Maudsley Card Sorting Test; FAS = Phonological Fluency Test; VF = Verbal Fluency Test; RFFT = Ruff Figural Fluency Test; WAIS-III = Wechsler Adult Intelligence Scale-Third Edition; RWT = Regensburger Verbal Fluency Test; DF = Design Fluency; TMT = Trail Making Test; TOVA = Test of Variable of Attention; TEA = Test of Everyday Attention; ST = Stroop Test; AGN = Affective Go-NoGo; PASAT = Paced Auditory Serial Addition Task; CFT = Complex Figure Test; DSST = Digit Symbol Substitution Test; CVLT = California Verbal Learning Test; COWAT = Controlled Oral Word Association Test; RAVLT = Rey Auditory Verbal Learning Test.

**Supplementary Table 3:** *Memory and learning*

| **Main Domain** | **Subdomain** | **Alternative Name** | **Definition** | **Tests** |
| --- | --- | --- | --- | --- |
| ***Short-term memory*** | Immediate memory | 1. Verbal Memory | Reproduction, recognition or recall of information directly or sometime after presentation | LMT, RAVLT, CVLT, WAIS-III, VRM, WMSR, WRM, GNT, DFDBT, TBT |
|  |  | 1. Visuo-spatial (non-verbal) memory | Allow information to be evaluated and perhaps stored longer through rehearsal and coding | SWM, SSP. DMS, PRM, PAL, BVRT, SRM, WMSR, RCFT, PASAT, WAIS-III |
| ***Long-term memory*** | 1. Explicit (declarative) memory | 1. Autobiographical, episodic or event memory | Records details salient to individuals life | PRM, SRM, CVLT, RAVLT, PAL, RCFT, WMSR |
|  |  |  | Needs conscious thinking ‘Knowing that’ | WAIS-III (Vocabulary) |
|  |  | 1. Semantic memory | Meaning of words and concepts or propositional knowledge (facts) | CFT, COWAT, GNT, WMSR, RBMT |
|  | 1. Implicit (non-declarative) or procedural memory | 1. Motor skill training | Does not need conscious thinking ‘Knowing how’ |  |
|  |  | 1. Priming or classical conditioning |  |  |

LMT = Logical Memory Test; CVLT = California Verbal Learning Test; WAIS-R/III = Wechsler Adult Intelligence Scale-Revised/Third Edition; VRM = Verbal Recognition Memory; WMSR = Wechsler Memory Scale Revisited; WRM = Word Recognition Memory; GNT = Graded Name Test; DFDBT = Digital Forward and Digital Backwards Test; TBT = Two Back Test; SWM = Spatial Working Memory; SSP = Spatial Span; RAVLT = Rey Auditory Verbal Learning Test; DMS = Delayed Matching to Sample; PRM = Pattern Recognition Memory; PAL = Paired Associated Learning; BVRT = Benton Visual Retention Test; SRM = Spatial Recognition Memory; RCFT = Rey Complex Figure Test; CFT = Complex Figure Test; PASAT = Paced Auditory Serial Addition Task; COWAT = Controlled Oral Word Association Test; RBMT = Rivermead Behavioural Memory Test.

| **Criteria** | Maurage et al., (2022) | Al-Mshari et al., (2020) | Bashir et al., (2017) | Li et al., (2017) | Bi et al., (2016) | Yuan et al., (2016) | Zhao et al., (2016) | Feng et al., (2015) | Jacobsen et al., (2005) |
| --- | --- | --- | --- | --- | --- | --- | --- | --- | --- |
| Research question or aim clearly stated and appropriate | Yes | Yes | Yes | Yes | Yes | Yes | Yes | Yes | Yes |
| Study population specified and defined | Yes | Yes | Yes | Yes | Yes | Yes | Yes | Yes | Yes |
| Includes sample size justification | Yes | Yes | No | No | No | No | No | No | No |
| Control selected from the same or similar population | Yes | Yes | Yes | Yes | Yes | Yes | Yes | Yes | Yes |
| Definitions, inclusion and exclusion criteria, algorithms, or processes to identify or select cases and controls are valid, reliable, and implemented consistently | Yes | Yes | Yes | Yes | Yes | Yes | Yes | Yes | Yes |
| Cases clearly defined and differentiated from controls | Yes | Yes | Yes | Yes | Yes | Yes | Yes | Yes | Yes |
| If less than 100 percent of eligible cases and/or controls, were selected for the study, cases and/or controls randomly selected from those eligible | NR | NR | NR | NR | NR | NR | NR | NR | NR |
| Use of concurrent controls | Yes | Yes | Yes | Yes | Yes | Yes | Yes | Yes | Yes |
| Investigators can confirm that the exposure/risk occurred before the development of the condition or event that defined a participant as a case | Yes | Yes | Yes | Yes | Yes | Yes | Yes | Yes | Yes |
| The measures of exposure clearly defined are valid, reliable, and implemented consistently across all study participants | Yes | Yes | Yes | Yes | Yes | Yes | Yes | Yes | Yes |
| The assessors of exposure/risk are blinded to the case or control status of participants | No | No | No | No | No | No | No | No | No |
| Statistical analysis accurate | Yes | Yes | Yes | Yes | Yes | Yes | Yes | Yes | Yes |
| **Quality rating (Good, Fair, and Poor)** | **Good** | **Good** | **Fair** | **Fair** | **Fair** | **Fair** | **Fair** | **Fair** | **Good** |

**Supplementary Table 4:**

*National Institutes of Health Study Quality Assessment Questionnaire*

**Supplementary Table 5:** *PRISMA Checklist*


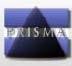
 **PRISMA 2020 Checklist**

| **Section and Topic** | **Item #** | **Checklist item** | **Location where item is reported** |
| --- | --- | --- | --- |
| **TITLE** | | |  |
| Title | 1 | Identify the report as a systematic review. | Title |
| **ABSTRACT** | | |  |
| Abstract | 2 | See the PRISMA 2020 for Abstracts checklist. | Abstract |
| **INTRODUCTION** | | |  |
| Rationale | 3 | Describe the rationale for the review in the context of existing knowledge. | Introduction |
| Objectives | 4 | Provide an explicit statement of the objective(s) or question(s) the review addresses. | Introduction |
| **METHODS** | | |  |
| Eligibility criteria | 5 | Specify the inclusion and exclusion criteria for the review and how studies were grouped for the syntheses. | Literature search |
| Information sources | 6 | Specify all databases, registers, websites, organisations, reference lists and other sources searched or consulted to identify studies. Specify the date when each source was last searched or consulted. | Literature search |
| Search strategy | 7 | Present the full search strategies for all databases, registers and websites, including any filters and limits used. | Literature search |
| Selection process | 8 | Specify the methods used to decide whether a study met the inclusion criteria of the review, including how many reviewers screened each record and each report retrieved, whether they worked independently, and if applicable, details of automation tools used in the process. | Analysis |
| Data collection process | 9 | Specify the methods used to collect data from reports, including how many reviewers collected data from each report, whether they worked independently, any processes for obtaining or confirming data from study investigators, and if applicable, details of automation tools used in the process. | Results |
| Data items | 10a | List and define all outcomes for which data were sought. Specify whether all results that were compatible with each outcome domain in each study were sought (e.g. for all measures, time points, analyses), and if not, the methods used to decide which results to collect. | Results |
|  | 10b | List and define all other variables for which data were sought (e.g. participant and intervention characteristics, funding sources). Describe any assumptions made about any missing or unclear information. | NA |
| Study risk of bias assessment | 11 | Specify the methods used to assess risk of bias in the included studies, including details of the tool(s) used, how many reviewers assessed each study and whether they worked independently, and if applicable, details of automation tools used in the process. | Analysis |
| Effect measures | 12 | Specify for each outcome the effect measure(s) (e.g. risk ratio, mean difference) used in the synthesis or presentation of results. | Results |
| Synthesis methods | 13a | Describe the processes used to decide which studies were eligible for each synthesis (e.g. tabulating the study intervention characteristics and comparing against the planned groups for each synthesis (item #5)). | Results |
|  | 13b | Describe any methods required to prepare the data for presentation or synthesis, such as handling of missing summary statistics, or data conversions. | Results, Quantitative analysis |
|  | 13c | Describe any methods used to tabulate or visually display results of individual studies and syntheses. | Results, Quantitative analysis |
|  | 13d | Describe any methods used to synthesize results and provide a rationale for the choice(s). If meta-analysis was performed, describe the model(s), method(s) to identify the presence and extent of statistical heterogeneity, and software package(s) used. | Analysis, Quantitative analysis |
|  | 13e | Describe any methods used to explore possible causes of heterogeneity among study results (e.g. subgroup analysis, meta-regression). | Analysis |
|  | 13f | Describe any sensitivity analyses conducted to assess robustness of the synthesized results. | NA |
| Reporting bias assessment | 14 | Describe any methods used to assess risk of bias due to missing results in a synthesis (arising from reporting biases). | NA |
| Certainty assessment | 15 | Describe any methods used to assess certainty (or confidence) in the body of evidence for an outcome. | NA |


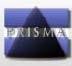
 **PRISMA 2020 Checklist**

| **Section and Topic** | **Item #** | **Checklist item** | **Location where item is reported** |
| --- | --- | --- | --- |
| **RESULTS** | |  |  |
| Study selection | 16a | Describe the results of the search and selection process, from the number of records identified in the search to the number of studies included in the review, ideally using a flow diagram. | Results |
|  | 16b | Cite studies that might appear to meet the inclusion criteria, but which were excluded, and explain why they were excluded. | Results |
| Study characteristics | 17 | Cite each included study and present its characteristics. | Results |
| Risk of bias in studies | 18 | Present assessments of risk of bias for each included study. | Analysis, Quantitative analysis |
| Results of individual studies | 19 | For all outcomes, present, for each study: (a) summary statistics for each group (where appropriate) and (b) an effect estimate and its precision (e.g. confidence/credible interval), ideally using structured tables or plots. | Qualitative analysis |
| Results of syntheses | 20a | For each synthesis, briefly summarise the characteristics and risk of bias among contributing studies. | Results |
|  | 20b | Present results of all statistical syntheses conducted. If meta-analysis was done, present for each the summary estimate and its precision (e.g.  confidence/credible interval) and measures of statistical heterogeneity. If comparing groups, describe the direction of the effect. | Quantitative Analysis |
|  | 20c | Present results of all investigations of possible causes of heterogeneity among study results. | Analysis, Quantitative Analysis |
|  | 20d | Present results of all sensitivity analyses conducted to assess the robustness of the synthesized results. | NA |
| Reporting biases | 21 | Present assessments of risk of bias due to missing results (arising from reporting biases) for each synthesis assessed. | Analysis, Quantitative Analysis |
| Certainty of evidence | 22 | Present assessments of certainty (or confidence) in the body of evidence for each outcome assessed. | Quantitative Analysis |
| **DISCUSSION** | |  |  |
| Discussion | 23a | Provide a general interpretation of the results in the context of other evidence. | Discussion |
|  | 23b | Discuss any limitations of the evidence included in the review. | Discussion |
|  | 23c | Discuss any limitations of the review processes used. | Discussion |
|  | 23d | Discuss implications of the results for practice, policy, and future research. | Discussion |
| **OTHER INFORMATION** | |  |  |
| Registration and protocol | 24a | Provide registration information for the review, including register name and registration number, or state that the review was not registered. | Methods |
|  | 24b | Indicate where the review protocol can be accessed, or state that a protocol was not prepared. | Methods |
|  | 24c | Describe and explain any amendments to information provided at registration or in the protocol. | NA |
| Support | 25 | Describe sources of financial or non-financial support for the review, and the role of the funders or sponsors in the review. | Funding |
| Competing  interests | 26 | Declare any competing interests of review authors. | Funding |
| Availability of data, code and other materials | 27 | Report which of the following are publicly available and where they can be found: template data collection forms; data extracted from included studies; data used for all analyses; analytic code; any other materials used in the review. | NA |
